# Analysis of Serum IgG1 to Predict Progression and Therapeutic Effect in Patients with Multiple Myeloma

**DOI:** 10.1155/2022/8628781

**Published:** 2022-03-17

**Authors:** Jingping Yin, Jun Qiu, Zheng Zhang, Bin Feng, Jinfang Shi, Dong Zheng

**Affiliations:** Center of Clinical Laboratory, The First Affiliated Hospital of Soochow University, Suzhou 215008, Jiangsu, China

## Abstract

**Objective:**

The correlation between laboratory indicators and clinical treatment effects and the prognosis of multiple myeloma remains poorly understood. Therefore, our study investigated whether serum IgG subclasses could be employed as potential indicators contributed to evaluate the therapeutic effect and prognosis of patients with multiple myeloma. *Patients and Methods*. Records of patients with multiple myeloma were initially diagnosed at the First Affiliated Hospital of Soochow University, China, from August 1, 2017, to February 28, 2020. The assessment abilities of serological indicators for therapeutic effect were evaluated in patients compared with healthy controls.

**Results:**

In 560 study patients with multiple myeloma, serum IgA, IgG, IgM, *κ*-LC, and *λ*-LC increased by15%, 33.04%, 1.96%, 27.50%, and 26.43%, respectively. Further analysis found that IgG1, IgG2, IgG3, and IgG4 were over the upper limit of the reference range with 26.38%, 6.09%, 8.12%, and 4.64%, respectively. *κ*-LC and *λ*-LC were found in the urine in 65.13% and 29.70%, respectively. In peripheral blood, the proportion of CD3^+^CD4^+^, CD3^−^CD19^+^ cells, and CD4^+^/CD8^+^ decreased, whereas CD3^+^CD8^+^ cells and CD16^+^/CD56^+^ increased, and the associated cytokines IL-2, IL-4, IL-6, TNF-*α*, and IFN-*γ* were upregulated in patients when compared with healthy controls. Furthermore, the serum levels of IgA, IgG, IgG1, IgG2, IgG3, and IgG4 gradually decreased in patients before, during, and after treatment. Similar results were found in serum and urine *κ*-LC and *λ*-LC.

**Conclusion:**

Serum IgG1 level could serve as the potential indicator for evaluating the therapeutic effect for patients with multiple myeloma. *κ*-LC and *λ*-LC also have the potential to be prognostic indicators. More studies are warranted to explore these serological indicators for personalized therapy in the future.

## 1. Introduction

Multiple myeloma (MM) is a common malignant tumor of the blood system characterized by abnormal proliferation of plasma cells [1,2]. Abnormal proliferation of plasma cells or myeloma cells in the bone marrow leads to bone destruction, and excessive secretion of monoclonal immunoglobulin inhibits normal synthesis of polyclonal immunoglobulin, leading to a series of clinical manifestations [1–3]. That is to say, multiple myeloma leads to multiple organ injuries, and patients eventually suffer from bone pain, fracture, renal insufficiency, anemia, bleeding, hypercalcemia, and susceptibility to infection, which are very complex and easy to be misdiagnosed. In recent years, under the application of bortezomib, thalidomide, Relidomide, and other targeted new drugs, overall survival (OS) and progression-free survival (PFS) of patients have been prolonged [4,5]. With the continuous extension of the curative effect and survival period, better biomarkers are needed to evaluate the treatment effect and prognosis of multiple myeloma and to provide better guidance for continuing treatment [6].

Multiple myeloma can be divided into the following eight types according to the increased type of abnormal immunoglobulin: IgG type, IgA type, IgD type, IgM type, IgE type, light chain type, dual clone type, and nonsecreted type, and two types according to the type of light chain: *κ* type, *λ* type [7]. However, the correlation among serum IgG, its subclasses, serum and urine *κ*-light chain (LC), *λ*-light chain (LC) levels, and the therapeutic effect and prognosis of multiple myeloma was not fully understood. In this study, we retrospectively analyzed 560 cases of hospitalized multiple myeloma patients from August 1, 2017, to February 28, 2020, in our hospital, and explored the potential biomarkers for evaluating the treatment effects of patients with multiple myeloma. Therefore, we hypothesized that total IgG level, levels of IgG subclasses, *κ*-LC, and *λ*-LC predict responsiveness to the therapeutic effect of multiple myeloma. To that end, we evaluated serum total IgG level, IgG subclasses, and serum and urine *κ*-LC and *λ*-LC in patients with multiple myeloma and healthy controls, and monitored total IgA, IgG, and IgG subclasses, *κ*-LC, and *λ*-LC responses to multiple myeloma patients before, during, and after treatment. Therefore, by analyzing the serological tests of multiple myeloma patients as well as the relationship between the examination indicators and the treatment effect, the aim is to find better biomarkers for the evaluation of treatment effects and prognosis of multiple myeloma.

## 2. Patients and Methods

### 2.1. Ethics Statement

The study was conducted in accordance with the Declaration of Helsinki and the Ethical Guidelines for Clinical Research. All serological testing and extractions of information from the database were approved by the Ethics Committee of the First Affiliated Hospital of Soochow University and performed in accordance with the relevant guidelines and regulations. All informed consent forms were signed by patients with multiple myeloma and healthy control.

### 2.2. Study Population and Samples

560 patients diagnosed with multiple myeloma at the First Affiliated Hospital of Soochow University, China, from August 1, 2017, to February 28, 2020, were prospectively enrolled in this study.

A 5 ml sample of peripheral blood was collected from enrolled patients with multiple myeloma and healthy controls for measurement of serum immunoglobulin and its subclasses, *κ*-LC and *λ*-LC, cytokine concentrations, and peripheral blood of patients with multiple myeloma was collected before, during, and after treatment. An additional 5 ml sample of peripheral blood was collected from patients and healthy controls for measurement of the proportion of CD3^+^CD4^+^, CD3^−^CD19^+^, CD4^+^/CD8^+^, CD3^+^CD8^+^, and CD16^+^CD56^+^ cells. A 10 ml sample of urine was collected from enrolled patients with multiple myeloma and healthy controls for measurement of *κ*-LC and *λ*-LC, and urine of some patients was collected before, during, and after treatment. The healthy controls were age and sex matched to patients with multiple myeloma.

### 2.3. Examination of Serum Immunoglobulin and Its Subclasses, *κ*-LC, *λ*-LC, Cytokine Concentration, and Urine *κ*-LC, *λ*-LC Concentration

Serum from patients with multiple myeloma and from healthy controls was extracted from fresh peripheral blood after centrifugation. Thereafter, serum IgA, IgG, IgM, *κ*-LC, and *λ*-LC levels were assessed by an automatic immunology analyzer (Beckman Image 800, CA, USA) following the method of scatter turbidimetry. IgG1, IgG2, IgG3, and IgG4 were detected with SIMENS BN II (Germany) following the method of scatter turbidimetry. Serum IL-2, IL-4, IL-6, TNF-*α*, and IFN-*γ* were measured with ELISA kits (Beyotime Biotechnology, China) according to the manufacturer's instructions. Urine *κ*-LC and *λ*-LC concentration were detected with an automatic immunology analyzer (Beckman Image 800, CA, USA) following the method of scatter turbidimetry.

### 2.4. Assessment of the Proportion of CD3^+^CD4^+^, CD3^−^CD19^+^, CD4+/CD8+, CD3^+^CD8^+^, and CD16^+^CD56^+^ Cells in Peripheral Blood

Peripheral blood from multiple myeloma patients and healthy controls was lysed with red blood cell lysis buffer. Thereafter, the proportion of CD3^+^CD4^+^, CD3^−^CD19^+^, CD4^+^/CD8^+^, CD3^+^CD8^+^, and CD16^+^CD56^+^ cells was analyzed by the BD Multitest™ IMK kit (Becton, Dickinson and Company, New Jersey, USA) using a flow cytometer (Becton, Dickinson and Company, New Jersey, USA) following the manufacturer's instructions.

### 2.5. Statistical Analysis

The data were presented as the mean ±SD. Each biological indicator was tested three times in this study. The unpaired Student's *t*-test was used for differences between the two groups. An ANOVA followed by the Newman–Keuls test was employed for multigroup comparisons. *P* values <0.05 were considered to indicate statistical significance for all statistical tests.

## 3. Results

### 3.1. Baseline Characteristics of Patients with Multiple Myeloma

Criteria for the diagnosis of multiple myeloma were fulfilled for 560 patients seen at the First Affiliated Hospital of Soochow University in Suzhou, China, from August 1, 2017, through February 28, 2020. In 560 patients, three percent of patients were younger than 40 years, and 10.71% were 70 years or older (Table 1); the median age was 59 years, and the range was 28–95 years (Table 1). Of these 560 patients, 59.46% were men (Table 1). The serum hemoglobin, creatinine, calcium, cholesterol, and triglyceride values are listed in Table 2. Anemia was present initially in 80.71% of patients, and a serum creatinine level of 73 mg/dL or more in 41.07%. Serum calcium levels were more than 2.52 mg/dL in 5.54% and less than 2.11 mg/dL in 19.11%. The cholesterol and triglyceride levels were increased by 21.96% and 21.79%, respectively.

### 3.2. Serum Levels of Immunoglobulin and Its Subclasses in Patients with Multiple Myeloma

To determine the immunoglobulin level in patients with multiple myeloma, we measured serum IgA, IgG, and IgM levels using chemiluminescence immunoassay. We found that serum IgA levels were 4.52 g/L or more in 15% and less than 0.82 g/L in 62.68%; serum IgM levels were more than 3.04 g/L in 1.96% and less than 0.46 g/L up to 68.04% (Table 3). However, serum IgG levels were more than 15.6 g/L in 33.04% and 36.43% were less than 7.51 g/L (Table 3). These results indicated that a higher proportion of patients with multiple myeloma were of the IgG type.

To further analyze IgG subclass levels in patients with multiple myeloma, we detected the serum levels of IgG1, IgG2, IgG3, and IgG4. We found that IgG2, IgG3, and IgG3 increased by 6.09%, 8.12%, and 4.64%, respectively, in patients with multiple myeloma. However, IgG1 was up to 26.38% (Table 3). These results showed that IgG1 was the main type in four IgG subclasses in multiple myeloma.

We also further analyzed the ratio of IgG subclasses and total IgG. The data showed that the IgG2/IgG ratio significantly decreased in patients compared with healthy controls (Table 3). But IgG1/IgG, IgG3/IgG, and IgG4/IgG ratios increased in patients with multiple myeloma compared with healthy controls, especially IgG4 (Table 3).

### 3.3. Level of *κ*-LC and *λ*-LC Increased in Serum and Urine of Patients with Multiple Myeloma

To analyze *κ*-LC and *λ*-LC levels in patients with multiple myeloma, we measured the *κ*-LC and *λ*-LC concentrations in serum and urine. In 560 patients, our results showed that *κ*-LC and *λ*-LC in serum were increased in patients with multiple myeloma by 27.50% and 26.43%; however, decreased by 45.00% and 42.86%, respectively (Table 4). A similar result was found in urine, *κ*-LC and *λ*-LC were increased in patients with multiple myeloma, especially *κ*-LC up to 65.13% (Table 4).

### 3.4. Proportion of CD3^+^CD4^+^, CD3^+^CD8^+^, CD3−CD19^+^, and CD16^+^CD56^+^ Cells and Associated Cytokines Concentration in Peripheral Blood of Patients with Multiple Myeloma

In order to further analyze the possible mechanism, we measured the proportion of CD3^+^CD4^+^, CD3^+^CD8^+^, CD3^−^CD19^+^, and CD16^+^CD56^+^ cells in peripheral blood of patients with multiple myeloma. In 57 patients with multiple myeloma, our results showed that the proportion of CD3^+^CD4^+^ cells and CD4^+^/CD8^+^ significantly decreased in peripheral blood compared with healthy controls (Table 5). Similarly, the proportion of CD3^−^CD19^+^ cells was decreased in most patients compared with healthy controls (Table 5). However, CD3^+^CD8^+^ and CD16^+^CD56^+^ cells, that were CTL cells and NK cells, significantly increased in peripheral blood of patients compared with healthy controls (Table 5).

We also further measured the immune cell-related cytokines in serum of patients with multiple myeloma. In serum of 57 patients, we found that IL-4, IL-6, IFN-*γ*, and TNF-*α* significantly increased in multiple myeloma compared with healthy control, especially IL-6 and IFN-*γ* (Table 6). However, IL-2 also increased in serum with *P* value <0.0762 (Table 6). In addition, C-reactive protein (CRP) significantly upregulated in serum of patients with multiple myeloma compared with healthy controls (Table 6).

### 3.5. Level of IgG Subclasses, *κ*-LC, and *λ*-LC Decreased in Patients with Multiple Myeloma after Treatment

To evaluate the role of IgA, IgG subclasses, *κ*-LC, and *λ*-LC in the treatment of multiple myeloma, we extracted a part of patients with over upper limit of reference range of testing items and analyzed the serum or urine levels of IgA, IgG, IgG1, IgG2, IgG3, and IgG4 and *κ*-LC and *λ*-LC in patients with multiple myeloma before, during, and after treatment. Our results showed that the levels of IgG and IgG1 gradually decreased before, during, and after treatment, and especially after treatment, they obviously decreased (Table 7). Similar results were found in IgA, IgG2, IgG3, and IgG4 in the processes of before, during, and after treatment (Table 7).

We also extracted patients with over the upper limit of the reference range and analyzed the serum and urine levels of *κ*-LC and *λ*-LC in patients with multiple myeloma before, during, and after treatment. The results showed that the serum levels of *κ*-LC and *λ*-LC gradually and significantly decreased before, during, and after treatment (Table 8). Similarly, the levels of *κ*-LC and *λ*-LC in urine gradually and significantly decreased before, during, and after treatment, especially after treatment compared with before treatment (Table 8).

## 4. Discussion

In our present study, the age and sex distributions of the 560 patients were similar to those in foreign studies of the 1027 patients with multiple myeloma seen at the Mayo Clinic [8] and a domestic study of the 304 patients with multiple myeloma seen at the Beijing Chaoyang Hospital [9]. However, in the current study, 10.71% of patients were 70 years or older, compared with 38% in the earlier foreign study. The incidence of multiple myeloma is much lower in the elderly population, and the lower percentage of patients 70 years or older in the current series is probably due to regional differences. In addition, some patients may not seek medical treatment due to differences in economic status, health concepts, and medical resources, so there are some differences in age distribution, especially among the elderly. This has yet to be proved. In this study, only 3.04% of patients were younger than 40 years at diagnosis, and this percentage is similar to foreign and domestic studies.

As expected, anemia was a major manifestation of myeloma and was present initially in 80.71% of patients. The mechanism in most patients is inadequate production of red blood cells due to either erythropoietin deficiency from accompanying renal failure or pronounced marrow replacement by myeloma cells [10–12]. The serum creatinine level was increased in 41.07% of our patients. The major causes of renal failure are myeloma kidney and hypercalcemia [13,14]. And dehydration and hyperuricemia are also reasons for renal failure [13–15].

Monoclonal immunoglobulin increases in the serum of patients with multiple myeloma, leading to dysfunction for the synthesis of normal polyclonal immunoglobulin, which makes it is easy to cause infection [16–18]. In the early stages of multiple myeloma, the sensitivity of immunoglobulin quantification is lower, and it is easy to miss the detection. However, in diagnosed patients, the determination of immunoglobulin content is helpful for observing the curative effect. In this study, the expression of the IgG type was the highest (33.04%) in patients with multiple myeloma, the expression of the IgA type (15%) was lower than that of the IgG type, and the expression of the IgM type (1.96%) was the lowest, which was consistent with the previous study. The other part of patients with multiple myeloma showed lower IgG, IgA, and IgM, owing to light chain type, nonsecreted type, IgD type, IgE type, etc. Most myeloma cells not only synthesize and secrete a large amount of monoclonal immunoglobulin but also have a imbalanced ratio of light and heavy chains. Serum light chain is one of the higher sensitive indicators for the clonal plasma cells in patients [19–21]. Our results showed that *κ*-LC and *λ*-LC significantly increased in the serum and urine of patients with multiple myeloma, consistent with previous studies. Serum and urine levels of light chain have high sensitivity and specificity, which are expected to be helpful for early detection and rapid diagnosis for patients with multiple myeloma via noninvasive detection.

The antitumor immune response of patients with multiple myeloma is dominated by cellular immunity [22]. T cell subsets play an important role in regulating the immune response and maintaining the immune stability in the body. In this study, CD3^+^CD4^+^ and CD4^+^/CD8^+^ ratios were significantly reduced, and CD3^+^CD8^+^ was increased in patients with multiple myeloma at the initial and progressive stages, which was consistent with the previous reports [23]. It can be seen that multiple myeloma patients have abnormal cellular immune regulation, and the immune function is closely related to the disease state. After effective treatment, CD3^+^CD4^+^, CD4^+^/CD8^+^ ratio, and CD3^+^CD8^+^ basically returned to normal. Therefore, by measuring T cell subsets in peripheral blood, the latest trends of the disease can be monitored. B lymphocytes are the major cells in the immune system that produce antibodies, present antigens, and secrete cytokines involved in immune regulation. Our data showed that B cells were significantly lower than healthy control in peripheral blood of patients with multiple myeloma, consistent with other researchers [23]. However, CRP may inhibit the T helper cells function, which would restrain IL-4 production, thereby interfering with polyclonal B cell activation [24]. It had reported that IL-6 production could be induced by TNF-*α* in a dose-dependent manner in myeloma cells [25]. NK cells can directly kill tumors and virus-infected cells. It plays an important role in the body's immune monitoring and early anti-infection immune process. Our results showed that NK cells were significantly higher than healthy controls in the initial stage, which was consistent with the report of Chan et al. [26]. It indicated that multiple myeloma patients still had the ability of immune self-stabilization in the early stage, and the NK cell function was significantly impaired in the progressive stage. Therefore, multiple myeloma patients have extensive immunodeficiency. Our research data indicate that there are abnormalities of CD3^+^CD4^+^, CD3^+^CD8^+^, CD3^−^CD19^+^, and CD16^+^CD56^+^ cells in patients with multiple myeloma. Whether these abnormalities are pathogenic factors of multiple myeloma (MM) or the result of the onset of MM. It is still poorly understood and needs to be further explored. In conclusion, lymphocyte subsets and related cytokines play an important role in the development of multiple myeloma, and monitoring these indicators can be used as potential biomarkers for the diagnosis of MM patients and monitoring treatment efficacy.

Several important prognostic factors were identified in our and other researcher's studies [8]. Most of these also have been identified as markers of high-risk disease in other studies [6]; thus, they are reliable and well-validated tools for counseling and patient care decisions. Many of the prognostic factors identified are simple clinical or laboratory variables such as age, hemoglobin, serum calcium, and serum creatinine values, all of which can be easily determined in all patients [27]. Although not analyzed in this study, other studies have shown that high lactate dehydrogenase levels [28], deletion of chromosome 13 [29,30], and circulating plasma cells [31] are other important adverse prognostic factors in multiple myeloma. Our study made interesting findings that IgG, IgG1, IgG2, IgG3, IgG4, *κ*-LC, and *λ*-LC, especially IgG1, *κ*-LC, and *λ*-LC, showed great changes in the process of treatment, that is, before, during, and after treatment. Therefore, these factors could be considered as prognostic factors, even as potential biomarkers for treatment effects for multiple myeloma.

## 5. Conclusion

In summary, by analyzing the serological and urine examinations of multiple myeloma patients as well as the correlation between the examination indicators and the treatment effect, we found that IgG1 expression was the highest in patients with multiple myeloma, and IgG1 changed greatly before, during, and after treatment. Therefore, IgG1 has great potential in predicting the progression and therapeutic efficacy of multiple myeloma patients. In this study, we also found that *κ*-LC and *λ*-LC, lymphocyte subsets, and related cytokines can be used to evaluate the therapeutic effect and prognosis of multiple myeloma.

This study, however, had some limitations. First, considerable numbers of patients with multiple myeloma did not detect all test items, so there is not sufficient data to support more accurate conclusions, such as IgG2 (*n* = 21), IgG3 (*n* = 28), and IgG4 (*n* = 16) in this study. Second, the correlation of IgG subclasses with overall survival (OS) and progression-free survival (PFS) was not shown, because there were not enough patients followed up. Therefore, more prospective studies from different medical centers are warranted to further characterize these factors for prediction and evaluation of the treatment effect and prognosis of multiple myeloma. The use of powerful, independent multiple prognostic factors in multiple myeloma has overcome the limitations of the Durie–Salmon staging system, which has been used for almost three decades as a staging and prognostic system for multiple myeloma.

## Figures and Tables

**Table 1:** Demographic data for 560 patients with multiple myelomas.

Factor	No. of patients	% of patients	Median	Range
*Age (y)*				
<40	17	3.04		
40–70	483	86.25		
>70	60	10.71		

Median			59	
Range				28–95

*Sex*				
Male	333	59.46		
Female	227	40.54		

**Table 2:** Laboratory test results in 560 patients with multiple myeloma.

	No. of patients	Range	Decreased N (%)	Normal N (%)	Increased N (%)
Hemoglobin (g/dL)	560	130–175	452 (80.71)	108 (19.29)	0
Creatinine (mg/dL)	560	41–73	36 (6.43)	294 (52.50)	230 (41.07)
Calcium (mg/dL)	560	2.11–2.52	107 (19.11)	422 (75.36)	31 (5.54)
Cholesterol (mg/dL)	560	<5.2	—	437 (78.04)	123 (21.96)
Triglyceride (mg/dL)	560	<1.7	—	438 (78.21)	122 (21.79)

**Table 3:** Concentration of serum monoclonal proteins in patients with multiple myeloma.

	Decreased N (%)	Normal N (%)	Increased N (%)
IgA (*N* = 560; range: 0.82–4.52 g/L)	351 (62.68)	125 (22.32)	84 (15)
IgG (*N* = 560; range: 7.51–15.6 g/L)	204 (36.43)	171 (30.54)	185 (33.04)
IgM (*N* = 560; range: 0.46–3.04 g/L)	381 (68.04)	168 (30.00)	11 (1.96)
IgG1 (*N* = 345; range: 4.05–10.11 mg/ml)	134 (38.84)	120 (34.78)	91 (26.38)
IgG2 (*N* = 345; range: 1.69–7.86 mg/ml)	196 (56.81)	128 (37.10)	21 (6.09)
IgG3 (*N* = 345; range: 0.11–0.85 mg/ml)	119 (34.49)	198 (57.39)	28 (8.12)
IgG4 (*N* = 345; range: 0.03–2.01 mg/ml)	31 (8.99)	298 (86.38)	16 (4.64)

**Table 4:** Concentration of serum and urine light chains in patients with multiple myeloma.

	Decreased N (%)	Normal N (%)	Increased N (%)
Serum *κ*-LC (*N* = 560; range: 629–1350 mg/dL)	252 (45.00)	154 (27.50)	154 (27.50)
Serum *λ*-LC (*N* = 560; range: 313–723 mg/dL)	240 (42.86)	172 (30.71)	148 (26.43)
Urine *κ*-LC (*N* = 543; range: <1.85 mg/dL)	—	189 (34.87)	353 (65.13)
Urine *λ*-LC (*N* = 543; range: <5 mg/dL)	—	381 (70.30)	161 (29.70)

**Table 5:** Proportion of immune cells in 57 patients with multiple myeloma compared with healthy controls.

	Healthy control	MM	*P* value
CD3^+^ (*N* = 57; range: 61.1–77%)	68.90 ± 4.58	68.93 ± 14.64	<0.99079
CD3^+^CD4^+^ (*N* = 57; range: 25.8–41.6%)	35.31 ± 4.58	28.95 ± 14.10	<0.02259
CD3^+^CD8^+^ (*N* = 57; range: 18.1–29.6%)	24.90 ± 3.25	38.82 ± 16.11	<0.001
CD4^+^/CD8^+^ (*N* = 57; range: 0.9–1.9)	1.41 ± 0.31	0.96 ± 0.74	<0.00290
CD3^−^CD19^+^ (*N* = 57; range: 7.3–18.2%)	11.04 ± 3.21	7.12 ± 8.15	<0.01654
CD16^+^CD56^+^ (*N* = 57; range: 8.1–25.6%)	13.53 ± 4.36	22.86 ± 13.95	<0.001

**Table 6:** Concentration of serum cytokines in patients with multiple myeloma.

	Healthy control	Multiple myeloma	*P* value
IL-2 (pg/ml, *N* = 57)	0.60 ± 1.23	2.08 ± 4.38	<0.0762
IL-4 (pg/ml, *N* = 57)	0.44 ± 0.83	2.13 ± 3.92	<0.0226
IL-6 (pg/ml, *N* = 57)	0.38 ± 0.64	10.18 ± 14.67	<0.0005
TNF-*α* (pg/ml, *N* = 57)	1.52 ± 1.39	4.08 ± 6.88	<0.0268
IFN-*γ* (pg/ml, *N* = 57)	0.44 ± 1.34	4.06 ± 7.16	<0.0075
CRP (*μ*g/ml, *N* = 560)	3.21 ± 1.81	47.67 ± 66.26	<0.0001

**Table 7:** Concentration of serum monoclonal proteins in patients with multiple myeloma before, during, and after treatment.

	Before treatment	During treatment	After treatment
IgA (*N* = 84)	28.80 ± 23.72	10.80 ± 11.98^*∗*^	9.90 ± 13.92^*∗*^
IgG (*N* = 185)	50.69 ± 33.24	17.43 ± 13.16^*∗*^	13.25 ± 10.19^*∗*^^#^
IgG1 (*N* = 91)	37.45 ± 21.30	11.71 ± 6.92^*∗*^	7.95 ± 3.54^*∗*^
IgG2 (*N* = 21)	17.11 ± 10.54	9.59 ± 5.45^*∗*^	7.6 ± 9.01^*∗*^
IgG3 (*N* = 28)	5.82 ± 7.38	0.84 ± 0.49^*∗*^	0.66 ± 0.39^*∗*^
IgG4 (*N* = 16)	35.51 ± 30.32	12.82 ± 18.92^*∗*^	13.87 ± 7.26^*∗*^

Compared with the before treatment group, ^*∗*^*P* < 0.05; compared with the during treatment group, ^#^*P* < 0.05.

**Table 8:** Concentration of serum and urine light chains in patients with multiple myeloma before, during, and after treatment.

	Before treatment	During treatment	After treatment
Serum *κ*-LC (*N* = 74)	5102.57 ± 5198.35	1580.39 ± 1214.76^*∗*^	1169.37 ± 963.99^*∗*^^†^
Serum *λ*-LC (*N* = 83)	3488.49 ± 3816.87	1099.27 ± 1070.41^*∗*^	761.48 ± 727.77^*∗*^^†^
Urine *κ*-LC (*N* = 164)	145.71 ± 526.10	33.50 ± 206.75^*∗*^	15.82 ± 90.29^*∗*^
Urine *λ*-LC (*N* = 86)	656.10 ± 1413.11	130.30 ± 473.83^*∗*^	168.07 ± 648.79^*∗*^

Compared with the before treatment group, ^*∗*^*P* < 0.05; compared with the during treatment group, ^†^*P* < 0.05.

## Data Availability

The data used to support the findings of this study are included within the article.
